# HER-2 positive breast cancer: decreasing proportion but stable incidence in Finnish population from 1982 to 2005

**DOI:** 10.1186/bcr2322

**Published:** 2009-06-18

**Authors:** Katri Köninki, Minna Tanner, Anssi Auvinen, Jorma Isola

**Affiliations:** 1 Institute of Medical Technology, University of Tampere, Biokatu 6, Tampere, 33014, Finland; 2 Department of Oncology, University Hospital of Tampere, Teiskontie 35, Tampere, 33520, Finland; 3 Tampere School of Public Health, University of Tampere, Medisiinarinkatu 3, Tampere, 33014, Finland

## Abstract

**Introduction:**

Classification of breast cancers according to the HER-2 oncogene status is of central importance in the selection of post-surgical therapies. A decrease in the proportion of HER-2-positive breast cancer has been suspected, but no data on the incidence trends at population level have been reported.

**Methods:**

We studied the proportion of HER-2-positive breast cancers by chromogenic in situ hybridization (CISH) in three cohorts (years 1982 to 1986 (n = 310), 1989 to 1992 (n = 108), and 2004 to 2005 (n = 713)) in the population of the Pirkanmaa hospital district (approximately 220,000 women). Cancer incidence rates were age-adjusted to the world standard population.

**Results:**

The proportion of HER-2-positive breast cancer declined from 21.6% (average in 1982 to 1986) to 13.6% (average in 2004 to 2005). However, during the same time period the age-adjusted incidence of all invasive breast cancers had increased by 40%. These opposite trends balanced each other and indicated that the incidence of HER-2-positive breast cancer has remained unchanged (Poisson regression coefficient for time trend 1.000; 95% CI = 0.989 to 1.012). In contrast, the incidence of HER-2-negative cancer showed 2% annual increase (Poisson regression coefficient 1.021, 95% CI = 1.016 to 1.026). Although HER-2-negative cancers were more likely to be diagnosed by mammography screening, the changes were more likely to be explained by etiological risk factors favoring HER-2-negative (and hormone receptor-positive) disease such as menopausal hormone therapy.

**Conclusions:**

These results document a significant decrease in the proportion of HER-2-positive breast cancer. However, the incidence of HER-2-positive cancer at the population level was found to be unchanged.

## Introduction

The human epidermal growth factor receptor-2 (HER-2) proto-oncogene has been in the focus of cancer research during the past two decades. It is well established that amplification of the HER-2 oncogene correlates with poor prognosis of patients [1]. More importantly, HER-2 oncogene is the molecular target of trastuzumab and lapatinib based therapies that are widely used in the treatment of HER-2-positive breast cancer. Currently all newly diagnosed breast cancers are assayed for HER-2 oncogene status [2-5]. HER-2-targeted therapies cause significant amounts of workload and costs, so accurate information on the incidence of HER-2-positive breast cancer is required to estimate the resources needed in clinics.

The incidence of HER-2 amplification in population-based cohorts of breast cancer is not known precisely. As reviewed by Cardoso and colleagues [6], the proportion of HER-2-positive tumors and/or overexpression has ranged between 10 and 40% in different studies. The figures are generally higher in older literature and in studies using immunohistochemical assays, which are considered to be less specific than assays based on *in situ *hybridization. Most studies describe relatively small cohorts of patients often including defined subtypes (node-negative, node-positive or *in situ *cancers). Due to the prognostic correlations studied, most studies describe patient populations whose breast cancers have been diagnosed 5 to 20 years earlier. It is well known that the overall incidence of breast cancer has increased significantly during the past decades [7,8]. Yet the time trends in the incidence of HER-2-positive breast cancer have remained unclear.

As a reference laboratory responsible for HER-2 tumor diagnostics, our laboratory has followed up the proportion of HER-2-positive tumors analyzed as part of our internal quality assurance. In this study we wanted to explore the apparent discrepancy between our current figures for HER-2 positivity (< 15%) and those previously reported in the literature (20 to 30%) [9,10]. Direct comparison between the current situation and the published studies was not possible, because the specificity and sensitivity of the HER-2 assay methods (immunohistochemistry vs. *in situ *hybridization) are likely to differ. Therefore, we studied the incidence trends of HER-2-positive breast cancer using chromogenic *in situ *hybridization (CISH) from 1131 samples of breast cancers diagnosed between 1982 and 2005 in a geographically defined hospital district, namely Pirkanmaa in Finland.

## Materials and methods

The study material consists of three population-based cohorts of breast cancer patients, diagnosed during the years 1982 to 1986 (n = 310), 1989 to 1992 (n = 108), and 2004 to 2005 (n = 713) in the Pirkanmaa Hospital District, Finland. All patients in the study had primary invasive breast cancer confirmed histopathologically. The patients diagnosed in 1982 to 1986 and 1989 to 1992 were selected randomly from the Finnish Cancer Registry database [11], which achieves close to 100% completeness [12]. Axillary lymph node status data were derived from the Finnish Cancer Registry, which was used to ascertain the similarity of the cohorts (Table 1). The cohorts comprised 32.4% and 11.0%, respectively, of all invasive breast cancer patients reported to the Cancer Registry from the study area. The newest cohort (cancers diagnosed between January 2004 and December 2005) included all invasive breast cancers submitted for hormone receptor and HER-2 analysis. This cohort consisted of 91.8% of cases reported to the Finnish Cancer Registry for this district.

**Table 1:** Characteristics of the study cohorts

**Patient cohort (year of primary diagnosis)**	**Female** population*	**Number of breast** cancers diagnosed per year**	**Number of breast cancers in the cohort** (n, %)**	**Age at diagnosis (median, range)**	**Proportion of axillary lymph node negative cancers**	**Proportion of estrogen receptor-positive cancers**	**Proportion of progesterone receptor-positive cancers**
1982 to 1986							
population-based**	215,639	191	957	63 (27 to 92)	53.1%	NA	NA
studied cohort			310 (32.4%)	59 (30 to 88)	56.6%	59.4%	69.7%
							
1989 to 1992							
population-based**,***	220,257	246	985	60 (28 to 92)	58.9%	72.0%	63.0%
studied cohort			108 (11.0%)	64 (34 to 88)	65.2%	77.9%	55.9%
							
2004 to 2005							
population-based**	231,000	389	777	NA	NA	NA	NA
studied cohort			713 (91.8%)	60 (25 to 94)	56.3%	89.8%****	77.3%

* Data from Statistics Finland (Pirkanmaa hospital district, Finland); ** Data from the Finnish Cancer Registry; *** Data from Laboratory of Cancer Biology, University of Tampere; **** The trend is statistically significant (*P *< 0.0001).

NA = not available.

Formalin-fixed and paraffin-embedded tumor samples were used for this study. Amplification of HER-2 was determined in all tumors by using CISH as previously described [13,14]. The cut-off for amplification was set at six gene copies per cell or the presence of a typical gene copy cluster [13]. The study was approved by the Ethical Committee at Tampere University Hospital.

The age-standardized breast cancer incidence was calculated using the World Health Organization (WHO) standard population [11]. Time trends in the proportion and incidence of HER-2-positive tumors were analyzed by regression methods, using a generalized linear model with binomial distribution and logarithmic link function and incidence trendswith Poisson regression, both in Stata 8.0 (StataCorp, College Station, Tx, USA). The outcome was a HER-2-positive tumor and the explanatory variable was the year of diagnosis as continuous variable. The exponentiated regression coefficient indicates average change in the proportion of HER-2-positive tumors per year relative to the first year analyzed. Statistical significance was assessed using a likelihood ratio test.

## Results

The proportion of breast cancers with HER-2 amplification was 21.6% in 1982 to 1986, 17.6% in 1989 to 1992, and 13.6% in 2004 to 2005 (Table 2). The tumor samples in the historical cohorts were selected randomly based on the cases reported to the Finnish Cancer Registry, and nearly all tumors (91.8%) were analyzed for the newest cohort (2004 to 2005), so these figures were used to estimate the incidence trends of HER-2-positive breast cancer in the population. The epidemiologic data (obtained from the Finnish Cancer Registry) showed that the annual number of newly diagnosed invasive breast cancers diagnosed in the Pirkanmaa Hospital District increased from 191 (average from 1982 to 1986) to 389 (average from 2004 to 2005) during the study period. The female population had increased only slightly (from 216,000 in 1982 to 1986 to 231,000 in 2004 to 2005). Using these figures, the average age-adjusted incidence of invasive breast cancer was found to have increased by 40% (56.3/100,000 in 1982 to 1986, 68.9/100,000 in 1989 to 1992, and 95.3/100,000 in 2004 to 2005; Tables 1 and 2). These figures are very close to those reported for the whole country [11].

**Table 2:** Trends in the incidence and proportion of HER-2-positive breast cancer from 1982 to 2005 in the Pirkanmaa Hospital District

**Breast cancer cohort**	Mean annual age-adjusted incidence* (per 100,000)	**Proportion of HER-2-positive breast cancers**	**Estimated age-adjusted incidence of HER-2-positive breast cancer** (per 100,000)**	**Estimated age-adjusted incidence of HER-2-negative breast cancer** (per 100,000)**
1982 to 1986	56.3	21.6% (67/310)	12.2	44.1
1989 to 1992	68.9	17.6% (19/108)	12.1	56.8
2004 to 2005	95.3	13.6% (97/713)	13.0	82.3

* Data from the Finnish Cancer Registry, age-adjusted to the world standard population; ** Estimated by multiplying the incidence of all breast cancers and the proportion of HER-2 positive.

HER-2 = human epidermal growth factor receptor 2.

**Figure 1:**
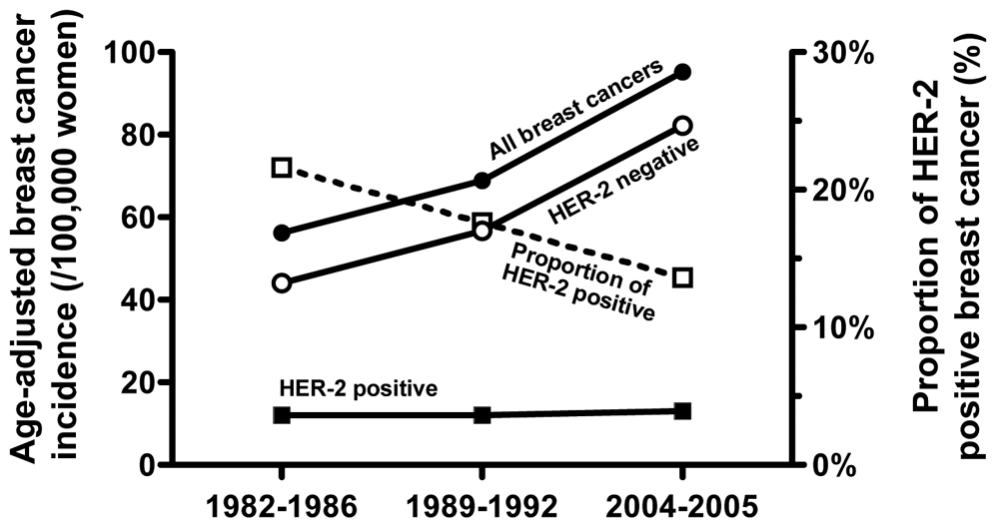
Time trends of age-adjusted breast cancer incidence and proportion of HER-2-positive breast cancer in the Pirkanmaan Hospital district from 1982 to 2005. Bullets = overall breast cancer incidence; rings = estimated incidence of human epidermal growth factor receptor 2 (HER-2)-negative breast cancer; closed squares = estimated incidence of HER-2-positive breast cancer; open squares = proportion of HER-2-positive breast cancer.

By multiplying the proportion of HER-2-positive breast cancer with the overall breast cancer incidence, we estimated that the age-standardized incidence of HER-2-positive breast cancer was 12.2/100,000 from 1982 to 1986, 12.1/100,000 from 1989 to 1992, and 13.0/100,000 from 2004 to 2005 (age-adjusted to WHO standard population; Table 2, 3rd column). In contrast, when multiplying the incidence of breast cancer with the proportion of HER-2-negative tumors (78.4%, 82.4%, and 86.4%, respectively), we found that the incidence of HER-2-negative cancer had almost doubled (from 44.1/100,000 to 82.3/100,000 women; Table 2). The rates are graphically presented in Figure 1. The Poisson regression analysis indicated a 2% annual increase in HER-2-negative cancer (incidence rate ratio = 1.021, 95% confidence interval (CI) = 1.016 to 1.026). For HER-2-positive cancer there was no trend (incidence rate ratio = 1.000, 95% CI = 0.989 to 1.012). Adjusting the results according to the age of the patients assayed for HER-2 did not affect the results.

The results of the most recent cohort were further analyzed for the possible effect of early diagnostics. We found that 33.4% of all invasive tumors were detected by screening mammography among the screened age groups (Table 3). Tumors that were HER-2 positive, estrogen receptor and progestrone receptor negative, or triple-negative were statistifically significantly underrepresented in patients whose cancers were detected by mammography screening (*P *= 0.039, *P *< 0.0001, *P *< 0.0001, and *P *= 0.100, respectively; Table 3).

**Table 3:** Characteristics of breast cancers diagnosed from 2004 to 2005 by the method of detection (screening vs. clinical)

	**Proportion detected by screening mammography (%)**	***P *value**
All patients *	196/587 (33.4%)	-
		
HER-2 negative	180/516 (34.9%)	
HER-2 positive	16/71 (22.5%)	0.039
		
ER positive	191/536 (35.6%)	
ER negative	5/51 (9.8%)	< 0.0001
		
PR positive	171/460 (37.2%)	
PR negative	25/102 (19.7%)	< 0.0001
		
Triple negative	4/32 (12.5%)	
Non-triple negative	192/555 (34.6%)	0.100

* Patients over 50 years old (age group of screening). ER = estrogen receptor; HER2 = human epidermal growth factor receptor 2; PR = progesterone receptor.

## Discussion

Our results indicate that the commonly used phrase '20% to 30% of breast cancers show HER-2 oncogene amplification' does not appear to be valid in a well-defined population-based cohort of Finnish breast cancer patients diagnosed in recent years. In our patient population, less than 15% of breast cancers showed amplification of the HER-2 oncogene. Thus, there is an apparent discrepancy with earlier literature [6], for which we sought to find an explanation by analyzing tumors from our archives retrospectively using the current CISH method. Comparison of the historical and more recent cohorts indicated that the proportion of HER-2-positive invasive breast cancer has clearly declined. The proportion found in the oldest cohort (1982 to 1986) was in good agreement with that reported in the literature, including a study from the same population [9], although our previous study was based on detection of HER-2 protein overexpression by immunohistochemistry instead of gene amplification [9].

As in most western countries, the age-adjusted incidence of all breast cancers had increased during the study period. In the Pirkanmaa Hospital District the increase in age-adjusted incidence was estimated to be 40%. Thus, we were able to disentangle the two opposing trends, that is, the decrease of the proportion of HER-2-positive breast cancer and increase in overall breast cancer incidence at the population level. These two trends were found to balance out each other. Our data indicated that the incidence of HER-2-positive breast cancer in the female population had remained stable. The increased incidence of breast cancer seemed to be due to HER-2-negative disease. To the best of our knowledge incidence trends in HER-2-negative and HER-2-positive breast cancers have not been reported in the literature.

Only relatively few studies have examined epidemiologic time trends of biologic subtypes of breast cancer. An increase in the incidence of hormone receptor-positive breast cancer has been documented [15-17]. As HER-2 and hormone receptors are inversely associated (e.g. *P *< 0.0001 in reference [6]; the p-value was calculated for our results in this study and the same phenomenon has been shown also in the reference 6), these observations are in line with our results of decreased incidence HER-2. Similar to the published reports, we also found a significant increase in the proportion of estrogen receptor-positive tumors in this study (from 59.4% in 1982 to 1986 to 89.8% in 2004 to 2005; Table 1). However, the data may be biased, because the estrogen receptor assay method had changed (ligand-binding assay was used in 1982 to 1986; immunohistochemistry on frozen sections in 1989 to 1992; immunohistochemistry on paraffin sections in 2004 to 2005). The sensitivity of these assay methods may differ and the possible bias is impossible to determine retrospectively.

In addition to hormone receptor data, changes in breast cancer histopathology has also been documented in the literature [18,19]. Increasing incidence of invasive lobular carcinoma [18,19] is in line with the decrease of HER-2-positive breast cancers, because these two features are inversely correlated [20,21]. Thus, based on the literature, the decreased proportion of HER-2-positive breast cancer may at least partly be explained by the increase of hormone receptor-positive and lobular carcinoma, which are mostly HER-2 negative.

Given the fact that the incidence of different subtypes of breast cancer really is changing, this suggests that the known risk factors of breast cancer do not affect all subtypes equally. As the incidence of HER-2-positive breast cancer was found to be constant, we can assume that the magnitude of its causative risk factors, which remain unknown, have probably remained unchanged. On the other hand, our results suggest that the impact of the risk factors for HER-2-negative cancer may have increased. One such risk factor could be menopausal hormone replacement therapy (HRT), which is a well-defined risk factor for breast cancer in general [22,23]. The use of HRT increased in Finland five-fold in the period from 1980 to 2000 [24], although a slight decrease occurred after 2003 [25]. However, the decrease in HRT use in Finland has been much smaller than in many other countries [25].

Several studies have demonstrated that the breast cancers in women who have used HRT are more frequently estrogen receptor and progesterone receptor positive than in those who have not used HRT [26,27]. Because it is well known that HER-2 amplification is inversely associated with hormone receptor positivity [6], it is possible that use of HRT may be associated with a risk of HER-2-negative disease. This theory is also supported by the association between HRT and increased incidence of invasive lobular breast carcinoma [28,29].

One possible explanation for the observed time trends is that intensified screening for breast cancer may detect a larger proportion of slowly growing HER-2-negative tumors with a longer lead-time than other tumor types. In line with previous studies that have characterized biomarker profiles of screen-detected breast cancers [30], our results demonstrated that HER-2-positive tumors are underrepresented in the screen-detected patient group, similar to that of tumors characterized by negative hormone receptor status or with a lack of all three markers (triple negative). In our study, screening is likely to give a partial explanation to the shift in biomarker profiles. Nationwide screening mammography was not in practice during the first period of the study (1982 to 1986), but was introduced gradually in 1989 to 1992; in 2004 to 2005 almost 90% of all women aged between 50 and 69 years participated in bi-annual mammography screening [31]. In the newest cohort (age group 50+ years) with clinically detected disease, the proportion of HER-2-positive disease (12.8%) was much lower than in early cohorts in 1982 to 1986 (19.1%, patients over 50 years old). This suggests that screening bias is a less important factor to explain the decrease seen in the proportion of HER-2-positive disease.

## Conclusions

These results document a significant decrease in the proportion of HER-2-positive breast cancer in an epidemiologically defined patient cohort during 1982 to 2005. At the same time, the overall incidence of breast cancer showed an increase of 40%. The estimated incidence of HER-2-positive cancer at the population level was found to be stable.

## Abbreviations

CI: confidence interval; CISH: chromogenic in situ hybridization; HER-2: human epidermal growth factor receptor-2; HRT: hormone replacement therapy.

## Competing interests

The authors declare that they have no competing interests.

## Authors' contributions

KK and JI were responsible for data collection, analysis, manuscript preparation, and editing. MT participated in data collection and designing of the study. AA contributed to study design, and planned and performed the statistical analysis. All authors read and approved the final manuscript.
